# Non-invasive Amino Acid Profiling of Embryo Culture Medium Using HPLC Correlates With Embryo Implantation Potential in Women Undergoing *in vitro* Fertilization

**DOI:** 10.3389/fphys.2020.00405

**Published:** 2020-05-20

**Authors:** Peng Huo, Yunshan Zhu, Chengqin Liang, Jun Yao, Jianghua Le, Linyuan Qin, Xiaocan Lei, Shun Zhang

**Affiliations:** ^1^School of Public Health, Guilin Medical University, Guilin, China; ^2^Department of Reproductive Medical Center, The Affiliated Hospital of Guilin Medical University, Guilin, China; ^3^College of Pharmacy, Guilin Medical University, Guilin, China; ^4^Clinical Anatomy & Reproductive Medicine Application Institute, Department of Histology and Embryology, University of South China, Hengyang, China

**Keywords:** amino acid, embryo development potential, *in vitro* fertilization, gemellary pregnancy, principal component analysis

## Abstract

This study aimed to determine the correlation between amino acid profiling of a 3-day-old embryo culture medium and embryo implantation potential in women undergoing *in vitro* fertilization (IVF). The data of 98 patients who received IVF treatment in our hospital from December 2015 to February 2017 were retrospectively analyzed. The 98 patients were grouped into a pregnant group (gemellary pregnancy), a non-pregnant group (non-pregnancy), and a blank control group. The amino acids from a 3-day-old embryo culture medium and blank control medium were collected and were analyzed using high performance liquid chromatography (HPLC). The HPLC results showed that amino acids including aspartate (ASP), serine (SER), glycine (GLY), histidine (HIS), taurine (TAU), arginine (ARG), threonine (THR), alanine (ALA), and proline (PRO) were detected in the 3-day-old embryo culture medium and blank control medium. There are significant differences between the pregnant group and non-pregnant group in peak height (H)-SER, surface area (S)-ASP, S-SER, S-HIS, and S-ALA. The discrimination analysis according to the peak height and peak area of amino acids revealed that the prediction rate of the pregnant group, non-pregnant group, and blank control group were 82.7, 95.7, and 100%. Further, by using the principal component analysis, we found that the prediction rate in these three groups were 90.4, 91.3, and 100%. Our data may suggest that using amino acid concentrations for principal component analysis and discriminant analysis has high accuracy in predicting the relationship between amino acid fingerprint and embryo implantation potential.

## Introduction

In recent years, infertility has become a worldwide medical problem affecting about 10–15% of people globally (Scoccia, 2015), while the incidence of infertility among people of reproductive age in China is 12.5–15% (Zhou et al., 2018). With the continuous advancement of medical research and technology, assisted reproductive technology (ART) has become the primary method for the treatment of infertility (Liberman et al., 2017). In ART, multiple pregnancies are the major concern, and the pregnancy complications include preterm delivery, low birth weight, and an increased risk for cerebral palsy. Reducing the incidence of multiple gestations while improving the pregnancy rates is an important focus on infertility research. Current embryo assessment strategies are largely based on embryo morphology (Rodgaard et al., 2015). However, the assessment on embryo morphology cannot reliably predict the embryo implantation potential. Though embryo transfer with two morphologically good embryos can produce relatively satisfying pregnancy rates, this can lead to 20–30% gemellary pregnancy, which threatens the health of mothers and babies. Therefore, development of an objective, accurate, fast, and affordable test that can aid in the assessment of embryo developmental potential for single embryo transfer is a significant aim of reproductive medicine.

Metabolomics, genomics, and proteomics are important components of system biology. Metabolomics can detect changes of metabolic substances in living organisms, which can reflect the intrinsic or extrinsic interactions of organisms, organs, and cells. Metabolites are intermediates or end products produced by organisms under specific conditions (Egea et al., 2014). In reproductive studies, the metabolic process of embryos can be determined by analyzing their related metabolites, which may provide a non-invasive method for predicting embryonic developmental capacity (Nel-Themaat and Nagy, 2011; Li et al., 2018). In the field of assisted reproduction, the development of embryos can be evaluated by detecting glucose, fatty acids, and amino acids in an embryo culture (Armstrong et al., 2018; De Rose et al., 2018). At present, the detection of amino acids can help to evaluate the development of embryos to a certain extent and help to judge the pregnancy outcome. Boyama et al. (2016) examined the relationship between the concentration of homocysteine (Hcy) in embryonic media and pregnancy outcomes, indicating that the concentration of Hcy is inversely related to pregnancy outcome (Borowiecka et al., 2012). With the deepening of research, amino acid metabolism, as a reference indicator for embryo evaluation, has been increasingly used in clinical work (Sturmey et al., 2008). The measurement of amino acid turnover has been suggested as non-invasive way to assess embryo viability (Sturmey et al., 2008). However, to date, how the changes of amino acid profiles during human embryo development affect pregnancy outcomes in IVF has not yet been determined.

In this study, we recruited 98 patients who had received IVF-embryo transfer (ET) treatment in our hospital and analyzed the concentrations of the amino acids in the embryo culturing medium by using high performance liquid chromatography (HPLC). By using principal component analysis (PCA), the relationship between amino acid fingerprint and embryo development potential was further explored. The present study may provide a relevant basis for the selection of high-quality embryos with high implantation potential during IVF by analyzing the amino acid fingerprint.

## Materials and Methods

### Study Subjects

The present study recruited 98 women who were infertile and were receiving IVF-ET treatment for pregnancy between December 2015 and February 2017 in the Reproductive Center of the Affiliated Hospital of Guilin Medical University. This study was approved by Ethics Committee of the Affiliated Hospital of Guilin Medical University (No. GLMC20183040), and written informed consent was signed by each patient. Inclusion criteria were: (1) age between 25–35 years old, (2) body mass index between 18–29 kg/m^2^, (3) primary infertile women with major indications for IVF, (4) women with fallopian tube problems and required IVF for pregnancy, (5) more than 5 oocytes were collected during IVF, and (6) the couples had no abnormalities in the chromosomes. Exclusion criteria were: (1) patients with a history of recurrent pregnancy loss; (2) patients with impaired ovulation; (3) patients with ovarian hyperstimulation syndrome; and (4) any significant systemic disease, endocrine, or metabolic disorder.

### Collection of Oocytes

Ovarian stimulation was performed using recombinant follicle stimulating hormone (FSH) with highly purified urinary gonadotropin using a luteal long protocol with a gonadotropin-releasing hormone (GnRH) agonist. When two leading follicles reached a mean diameter of ≥18 mm or three leading follicles reached a mean diameter of ≥16 mm, 5000–10000 IU of recombinant human chorionic gonadotropin (hCG) was injected. Ultrasonography-guided transvaginal oocyte retrieval was performed 36 h after hCG administration.

### Sperm Collection

Semen was collected on the day of oocyte retrieval according to the standard protocols of IVF. Following liquefaction, the whole semen was mixed with the G-IVF plus solution (Vitrolife, Sweden). The sperm was allowed to swim-up for 40 min at 37°C in an incubator with 6% CO_2_. The supernatant with swim-up sperms were collected and centrifuged at 200 *g* for 5 min. The sperm pellet was re-suspended in 1.0 ml G-IVF plus solution and the resuspended pellet was later used for IVF/intracytoplasmic sperm injection (ICSI).

### ET and Culture Medium Collection

The fertilization was performed using IVF/ICSI, and the embryos were transferred 3 days after oocyte retrieval. Embryo quality was evaluated by morphological criteria based on the degree of fragmentation and the regularity of blastomeres on day three after fertilization. The embryos were graded as follows: grade A, 0% anucleate fragments, regularity of blastomeres, and no apparent morphological abnormality; grade B, <20% anucleate fragments, regularity of blastomeres, and no apparent morphological abnormality. The embryos with grade A or grade B were included in the study. The culture medium for embryos at D3 after embryo transfer was collected. For the blank control group, the culture medium without embryos culturing were used in this study. All the collected medium was stored in liquid nitrogen before amino acids analysis.

### Follow-Up Study

Clinical pregnancy was defined as the positive hCG test from urine at 14 day after ET and the presence of one or more gestational sacs at 28 day after ET. Miscarriage was defined as pregnancy loss before 12 weeks of gestation. In the study, a total of 52 patients who had a gemellary pregnancy were defined as being in the pregnant group, and the 46 patients with two embryos but who had lost their pregnancy before 12 weeks of gestation were defined as the non-pregnant group.

### Solvents, Reagents and HPLC Analysis

All solvents used were of HPLC grade and were purchased from Sigma-Aldrich (St. Louis, United States). The 18 individual amino acid standards, including histidine (His), serine (Ser), arginine (Arg), glycine (Gly), aspartate (Asp), glutamate (Glu), threonine (The), alanine (Ala), proline (Pro), cystine (Cys), lysine (Lys), tyrosine (Tyr), methionine (Met), valine (Val), isoleucine (Ile), leucine (Leu), glutamine (Gln), and phenylalanine (Phe), were purchased from Sigma-Aldrich (St. Louis, United States). Sample preparation from culture media was performed using the Water AccQ-Tag^TM^ Ultra Derivatisation Kit (Waters, Milford, United States). Firstly, the derivatization reagent (AccQ-Tag Ultra Reagent power, AQC) was reconstituted in AccQ-Tag Reagent Diluent (acetonitrile). Standard sample mixtures (10 μL) or the culture media (10 μL) from the samples was mixed with 20 μL of derivatization and 70 μL of AccQ-Tag Ultra Borate Buffer in a tapered glass vial. The mixture was vortexed twice with a 1 min interval standing at room temperature and incubated in a heating block for 10 min at 55°C. Chromatographic separation was achieved by injection of 1 μL of sample or standard sample using an ACCQ-TAG ULTRA C18 1 μm, 2.1 × 100 mm column at 37°C using a gradient mobile phase consisting of 100% HPLC-grade water (A), 100% AccQ-Tag Ultra Eluent A (B), idle (C), and 100% HPCL-grade acetonitrile (D). Gradient conditions are shown in Table 1. A Tunable UV detector was used to detect sample absorbance at 260 nm in single wavelength mode. Data acquisition and processing was performed using Waters Empower 2 chromatography software using the manufacturer-defined method for protein hydrolysates. Individual amino acid elution times were manually verified in standard samples prior to processing with the manufacturer defined methodology to quantitate amino acids. The peak height (H) and surface area (S) for corresponding amino acids were extracted and was exported to Excel.

**TABLE 1:** Chromatographic gradient elution profile for amino acid analysis.

**Time (min)**	**A (%)**	**B (%)**	**C (%)**	**D (%)**	**Flow (ml/min)**
0.00	0.0	0.0	0.0	0.0	1.00
11.00	0.0	100.0	0.0	0.0	1.00
11.01	0.0	99.0	0.0	1.0	1.00
21.00	0.0	95.0	0.0	5.0	1.00
22.00	0.0	91.0	0.0	9.0	1.00
28.50	0.0	83.0	0.0	17.0	1.00
38.00	0.0	83.0	0.0	17.0	1.00
38.01	40.0	0.0	0.0	60.0	1.00
41.00	40.0	0.0	0.0	60.0	1.00
41.01	0.0	100.0	0.0	0.0	1.00
68.00	0.0	100.0	0.0	0.0	1.00
68.01	40.0	0.0	0.0	60.0	1.00
100.00	40.0	0.0	0.0	60.0	1.00

### Statistical Analysis

All the data were analyzed using the SPSS 20.0 software (IBM, Armonk, United States). The data were presented as mean ± standard deviation. Significant differences between two groups were analyzed using an unpaired *t*-test. *P* < 0.05 was considered statistically significant.

## Results

### General Characteristics of the Recruited Patients

In the present study, 98 patients were recruited in our analysis, and 52 patients were classified into the pregnant group and 46 patients were classified into the non-pregnant group. The general characteristics of all the patients were shown in Table 2 and there was no significant difference in these variables between the pregnant group and non-pregnant group (Table 2).

**TABLE 2:** Clinical parameters of the recruited subjects.

**Variables**	**Gemellary**	**Non-**	***P* values**
	**pregnancy**	**pregnancy**	
Age (years)	29.53 ± 4.52	30.04 ± 4.56	0.609
Duration of infertility (years)	3.1 ± 1.97	3.17 ± 2.20	0.874
Body mass index (kg/m^2^)	21.84 ± 3.00	21.90 ± 2.92	0.916
Baseline FSH levels (IU/L)	6.22 ± 1.25	5.96 ± 1.47	0.404
Baseline LH levels (IU/L)	5.48 ± 2.03	4.71 ± 1.88	0.071
Baseline E2 levels (pg/mL)	141.52 ± 47.48	132.42 ± 57.27	0.447
Initial dose of gonadotropin (IU)	194.17 ± 59.25	186.59 ± 54.92	0.540
FSH levels on the day of starting gonadotropin administration (IU/L)	3.46 ± 1.033	3.345 ± 1.41	0.709
LH levels on the day of starting gonadotropin administration (IU/L)	1.57 ± 0.81	1.62 ± 0.70	0.730
E2 levels on the day of starting gonadotropin administration (pg/ml)	54.11 ± 35.96	50.31 ± 35.69	0.628
Progesterone levels on the day of starting gonadotropin administration (IU/L)	1.70 ± 0.76	1.64 ± 0.61	0.682
LH levels on the day of HCG administration (IU/L)	2.28 ± 1.86	1.85 ± 1.02	0.140
E2 levels on the day of HCG administration (ng/mL)	15.18 ± 7.93	17.13 ± 7.49	0.248
Progesterone levels on the day of HCG administration (IU/L)	3.03 ± 0.89	3.02 ± 0.89	0.971
Endometrial thickness on the day of embryo implantation	10.26 ± 2.02	10.70 ± 2.38	0.374

### Construction of Standard Curve for Determination of Amino Acid Concentrations

The concentrations of 0.1, 0.2, 0.3, 0.4, and 0.45 μmol/ml for amino acid standards were used to construct the standard curve. The construct standard curves were shown in Table 3.

**TABLE 3:** Standard curve construction for amino acids.

	**0.1 μmol/ml**	**0.2 μmol/ml**	**0.3 μmol/ml**	**0.4 μmol/ml**	**0.45 μmol/ml**	**Standard curve**	**R square**
ASP	142.4	183.1	223.6	265.5	289.7	*Y* = 416.67× + 99.704	0.9994
SER	131.9	195.7	260.5	322.1	347.7	*Y* = 620.88× + 71.235	0.9992
GLU	138.8	167	196.1	223	234.7	*Y* = 275.11× + 111.85	0.9992
GLY	106.1	175.2	248.3	313.6	344	*Y* = 684.05× + 39.066	0.9993
HIS	108.7	188.5	259.3	331.6	363.5	*Y* = 726.96× + 39.501	0.9991
ARG	173.3	255.2	329.5	398.8	436.2	*Y* = 744.63× + 102.66	0.999
THR	121.5	221	320.1	415.2	456.5	*Y* = 926.66× + 27.689	0.9994
ALA	165.6	229.4	287.1	344.3	375.4	*Y* = 593.8× + 108.15	0.9993
PRO	139	233.3	325	412	457.2	*Y* = 906.28× + 50.479	0.9998
CYS	112.3	200.1	282.1	361.6	405	*Y* = 829.9× + 31.55	0.9995
TYR	138.9	262	379.8	506	565.5	*Y* = 1219.1× + 16.898	0.9999
VAL	128	216.2	299.1	375.9	420.12	*Y* = 826.6× + 48.15	0.999
MET	131	240.1	344.2	440.2	495.2	*Y* = 1031.6× + 30.955	0.9995
LYS	215.6	309.2	404.3	487.2	525.9	*Y* = 889.83× + 129.64	0.9993
ILE	142	240.2	336.2	423.1	474	*Y* = 940.34× + 49.612	0.9998
LEU	138.2	233.2	317.1	405.7	450.9	*Y* = 885.89× + 51.368	0.9994
PHE	132.9	249.2	381.8	505.7	567.6	*Y* = 1250.2× + 3.8203	0.9999
TAU	179.5	258.4	330.5	395.8	448.5	*Y* = 67.54× + 119.92	0.9945

### Detection of Amino Acids in the Culture Medium

In the present study, eight amino acids including SER, GLY, HIS, TAU, ARG, THR, ALA, and PRO were detected in the culture medium from the blank control group, non-pregnant group, and pregnant group. The peak height and surface area of the HPLC results for amino acids were all determined (Table 4).

**TABLE 4:** Concentrations of amino acids in the recruited subjects.

	**Pregnant**	**Non-pregnant**	***P* values**
S-ASP	107.71 ± 36.12	126.31 ± 45.58	0.028
S-SER	124.16 ± 43.27	42.56 ± 24.52	0
S-GLY	88.46 ± 60.34	86.35 ± 50.44	0.853
S-HIS	130.23 ± 37.54	80.74 ± 100.57	0.001
S-TAU	37.63 ± 15.63	34.72 ± 10.30	0.287
S-ARG	46.49 ± 17.64	41.15 ± 14.91	0.112
S-THR	82.64 ± 34.62	85.78 ± 27.57	0.625
S-ALA	118.12 ± 63.09	153.90 ± 42.21	0.002
S-PRO	41.06 ± 20.10	47.74 ± 18.75	0.095
H-ASP	6.28 ± 2.06	5.79 ± 2.65	0.315
H-SER	8.99 ± 2.83	3.63 ± 2.37	0
H-GLY	7.01 ± 2.13	6.27 ± 3.99	0.256
H-HIS	5.14 ± 3.04	4.99 ± 3.10	0.812
H-TAU	3.02 ± 1.43	2.94 ± 1.07	0.767
H-ARG	3.96 ± 1.48	3.88 ± 1.69	0.805
H-THR	8.80 ± 4.10	8.69 ± 3.01	0.884
H-ALA	11.47 ± 5.69	11.73 ± 3.11	0.783
H-PRO	3.78 ± 1.50	3.59 ± 1.12	0.47

### Discriminant Analysis Using Amino Acid Data

**FIGURE 1:**
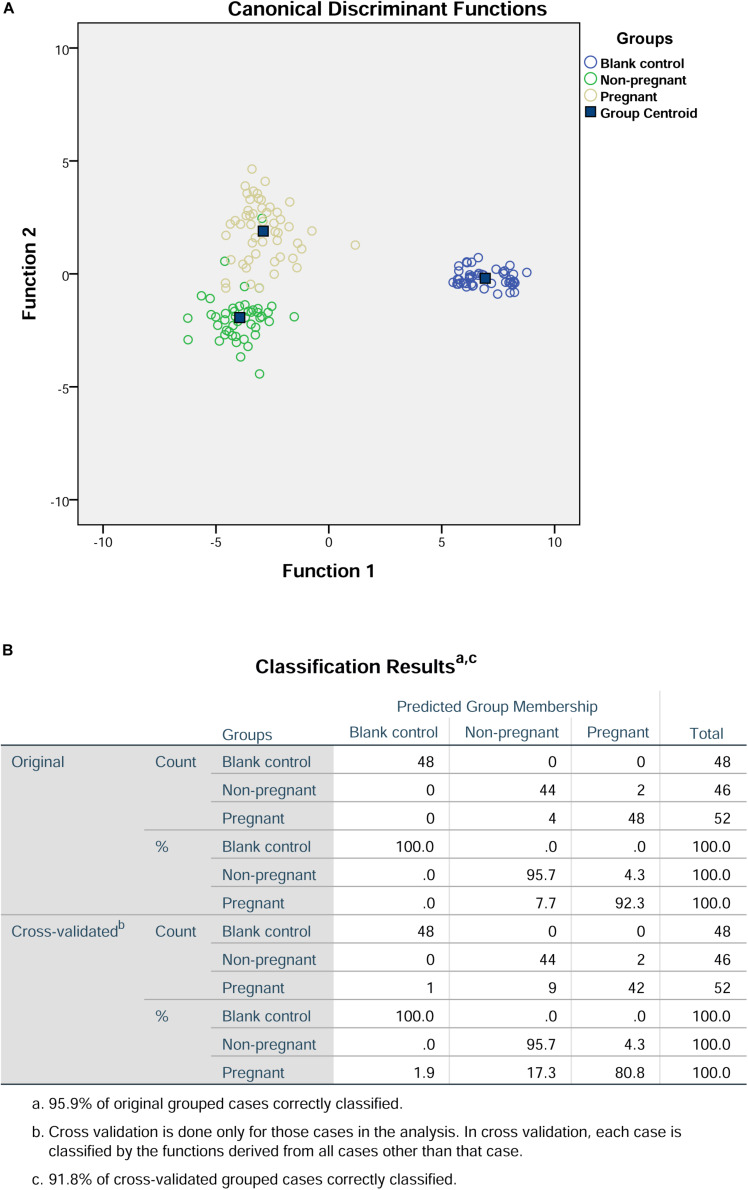
Discriminant analysis of amino acid profiles in the culture medium. **(A)** Canonical discriminant functions. **(B)** Classification results.

First of all, we included the values for peak height and surface area of the detected amino acids for the discriminant analysis. The results of the discriminant analysis were shown in Figure 1. The cross-validation results showed that the prediction rate for the blank control group, non-pregnant group, and pregnant group were 100, 95.7, and 80.8%, respectively. In addition, the overall prediction rate is 95.9%, and the accuracy of the cross-validation is 91.8%.

**FIGURE 2:**
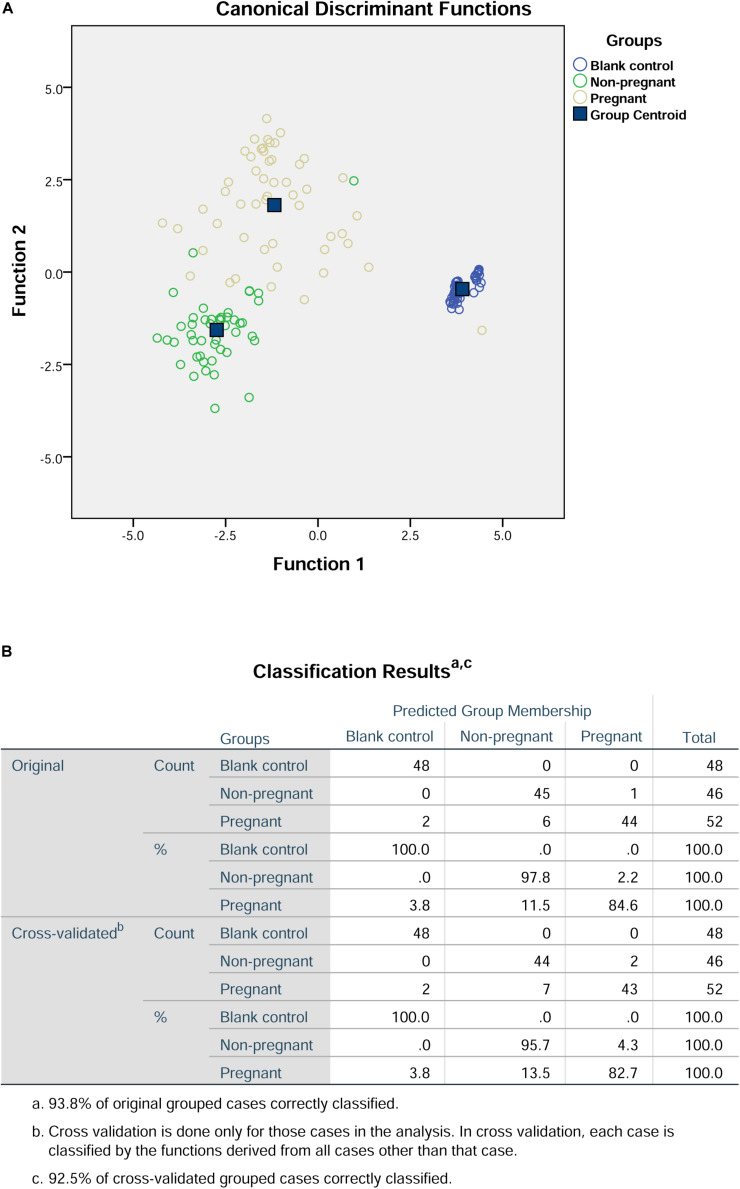
Discriminant analysis of S-ASP, S-SER, S-HIS, S-ALA, and H-SER in the culture medium. **(A)** Canonical discriminant functions. **(B)** Classification results.

In terms of the peak height and surface area, the values of S-SER, S-HIS, and H-SER were significantly higher in the pregnant group than that in the non-pregnant group, while the values of S-ASP and S-ALA were significantly lower in the pregnant group than that in the non-pregnant group (Table 4). In this regard, we included S-ASP, S-SER, S-HIS, S-ALA, and H-SER to perform the discriminant analysis. The results of the discriminant analysis were shown in Figure 2. The cross-validation results showed that the prediction rates for the blank control group, non-pregnant group, and pregnant group were 100, 95.7, and 82.7%, respectively. The overall prediction rate is 93.8%, and the accuracy of the cross-validation is 92.5%.

### PCA in Combination With Discriminant Analysis for Amino Acid Date

**FIGURE 3:**
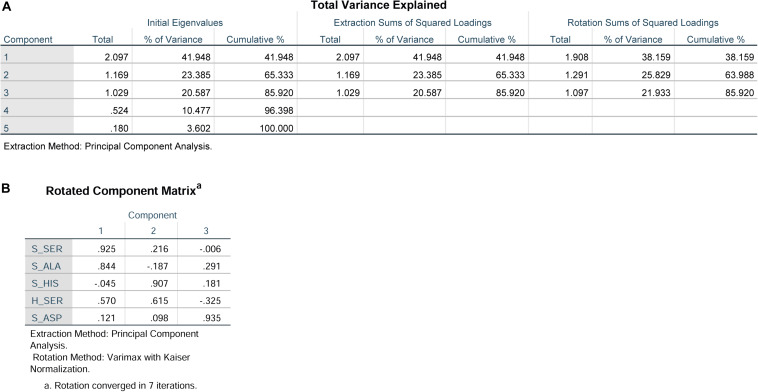
Principal component analysis of S-ASP, S-SER, S-HIS, S-ALA, and H-SER. **(A)** Total variance explained. **(B)** Rotated component matrix.

Principal component analysis was used to reduce the dimensionality of the dataset and provide an objective amalgamation of the amino acid data. As determined from PCA using included S-ASP, S-SER, S-HIS, S-ALA, and H-SER data, three principle components were extracted (Figure 3A), and the formulas were: F1 = 0.121S-ASP + 0.925S-SER-0.045S-HIS + 0.884 S-ALA + 0.570 H-SER; F2 = 0.098S-ASP + 0.216 S-SER + 0.907 S-HIS-0.187 S-ALA + 0.615 H-SER; F3 = 0.935 S-ASP-0.006 S-SER + 0.181 S-HIS + 0.291 S-ALA-0.325 H-SER (Figure 3B).

**FIGURE 4:**
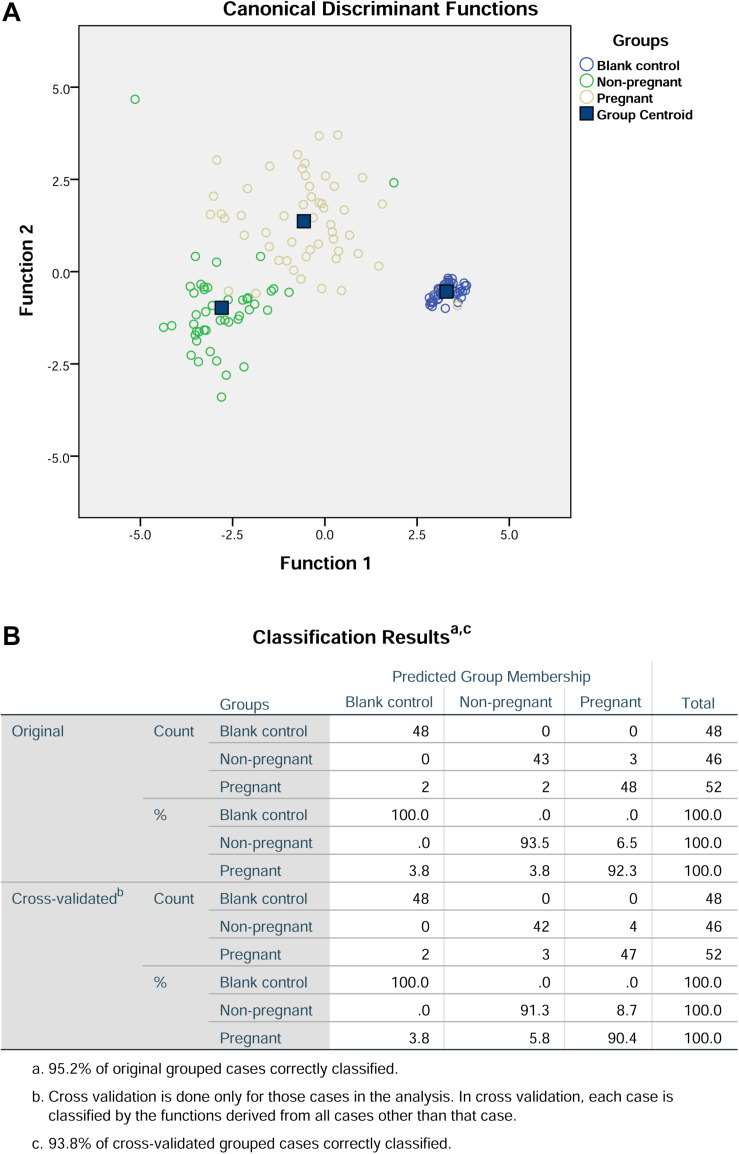
Discriminant analysis of the three components from principal component analysis. **(A)** Canonical discriminant functions. **(B)** Classification results.

Further discriminant analysis using the three principle components showed that the prediction rate for the blank control group, non-pregnant group, and pregnant group were 100, 91.3, and 90.4%, respectively (Figure 4). In addition, the overall prediction rate in this prediction model is 95.2%, and the accuracy of the cross validation is 93.8% (Figure 4).

## Discussion

In the present study, we determined the concentrations of amino acids in D3 embryo culture medium. ASP, SER, GLY, HIS, TAU, ARG, THR, ALA, and PRO were detected in the culture medium. There were significant differences between the pregnant group and non-pregnant group in H-SER, S-ASP, S-SER, S-HIS, and S-ALA. The discrimination analysis according to the peak height and peak area of amino acids revealed that the prediction rate of the pregnant group, non-pregnant group, and blank control group are 82.7, 95.7, and 100%. In addition, by using the principal component analysis, we found that the prediction rate in these three groups were 90.4, 91.3, and 100%. Our data may suggest the discrimination model derived from data (amino acid concentrations) for PCA and discriminant analysis has high accuracy in predicting the relationship between amino acid fingerprint and embryo implantation potential.

With the continuous development and improvement in ART, the evaluation of embryonic development potential has been given more and more attention. From a morphological point of view, low-quality embryos have serious morphological abnormalities such as cytoplasmic coarse particles, intracytoplasmic inclusion bodies, or vacuoles. The morphological scores of the pronuclear stage of the embryo mainly include the size, number, and location of the pronucleus and the morphology of cytoplasm (Fragouli et al., 2014). The morphological scores of the cleavage stage mainly include the number and symmetry of the blastomeres, the extent of the fragments, and whether there are vacuoles (Fragouli et al., 2014). Studies have shown that the degree of fragmentation in the embryo on D3 is closely related to the successful rate of embryo implantation (Scott and Smith, 1998). Embryologists have observed that when the fragment rate in the embryo under the microscope is <10%, its influence can be ignored Alpha Scientists in Reproductive Medicine and ESHRE Special Interest Group of Embryology (2011). However, high fragmentation rates can severely reduce the successful rates of embryo implantation. At the same time, when the fragmentation rate of the embryo is increased from 0 to 35%, the chromosome translocation rate can be increased by 10–30% Alpha Scientists in Reproductive Medicine and ESHRE Special Interest Group of Embryology (2011). However, the generation and migration of debris is dynamic, so the degree of fragmentation of embryos has not been used as an independent indicator to evaluate the potential of embryo implantation (Athayde Wirka et al., 2014). As women older than 35 years showed decreased function of their ovaries with an increased aneuploidy rate of eggs American College of Obstetricians and Gynecologists Committee on Gynecologic Practice and Practice Committee (2014), these will lead to unreliable morphological evaluation of the embryo. As such, we only included the patients with an age less than 35 for the amino acid profile analysis in the embryo culture medium.

Metabolomics is a new discipline established in the 1990s and is another important component of systems biology following genomics, transcriptomics, and proteomics (Fiehn, 2002; Garlow, 2002; Heijne et al., 2003; Gilchrist et al., 2006; Kern et al., 2007). Amino acid metabolism plays a very important role in early embryonic growth and development (Wu, 2010). The synthesis of proteins required for embryonic growth and development is closely related to amino acid metabolism. Among them, glutamic acid is formed by amino acid transferase, and further decomposes to produce α-ketoglutaric acid, which forms adenosine triphosphate under the action of a citric acid cycle. In addition, glutamate provides a secondary source of carbon and nitrogen for the re-synthesis of pyrimidines and hydrazines, and acts as a reducing agent to protect cells from oxidative stress. Arginine forms nitric oxide through the catalytic action of nitric oxide synthase and is involved in the signal transduction pathway of embryos, which is an essential metabolic pathway for embryonic growth and development (Wu, 2010). Recently, metabolomics have also been applied in ART research. Boyama et al. (2016) tested the relationship between the concentration of homocysteine (Hcy) in the culture medium and the pregnancy outcome, indicating that the concentration of Hcy is inversely related to the pregnancy outcome (Boyama et al., 2016). Montsko et al. (2015) used a high performance liquid chromatography-mass spectrometry to analyze the composition of embryo culture medium and found that the amount of conjugated globin-α1 fragment was correlated with the outcome of pregnancy during IVF, suggesting that detection and quantitation of the α-1 haptoglobin fragment of the culture medium proved to be a useful additional method for identifying non-viable embryos. Lydie et al. studied metabolic profiling of spent embryo culture media using high-resolution nuclear magnetic resonance, and their data showed that embryos with poor development potential consumed more amino acids including proline, threonine, lysine, methionine, tyrosine, and phenylalanine (Nadal-Desbarats et al., 2013). Seli et al. (2008) showed that glutamate concentrations determined by1H NMR were significantly higher in a spent culture media of embryos that resulted in pregnancy and delivery compared to those that failed to implant, and proton NMR spectroscopy predicted the viability of individual embryos with a sensitivity of 88.2% and a specificity of 88.2%. Brison et al. (2004) used HPLC to detect the concentration of amino acids in an embryo culture medium at 24 h, and found that the turnover of three amino acids, Asn, Gly, and Leu, was significantly correlated with a clinical pregnancy and live birth. These correlations were independent of known predictors, such as female age, basal levels of FSH, embryo cell number, and embryo morphological grade (Brison et al., 2004). Subsequently, Stokes et al. (2007) used the HPLC to analyze the post-thaw amino acid metabolism of human embryos from day two to day three of development and showed a significant difference in the utilization of glutamine, alanine, glycine, glutamate, arginine, and lysine between the thawed embryos which developed to the blastocyst stage and those which arrested prior to blastocyst formation. In addition, this method predicts with 87% accuracy which frozen-thawed embryo would develop to the blastocyst stage (Stokes et al., 2007). In our experiments, we showed that SER, ASP, HIS, and ALA in the culture medium showed a significant difference between the pregnant group and non-pregnant group. Based on the above evidence, the changes of amino acid profiles were varied among different studies, suggesting the dynamic changes of amino acid metabolism during embryo development. In our study, PCA was used to reduce the dimensionality of the dataset and provide an objective amalgamation of the amino acid data. The results showed that the prediction rate in these three groups were 90.4, 91.3, and 100%, suggesting good predictive accuracy in our study. However, whether changes in the concentrations of these amino acids will lead to changes in the expression of proteins that may be related to embryo development is unknown in the present study. Future studies may be performed to determine the possible mechanistic correlation between amino acids profiles and embryo development potential.

However, in our study, several limitations should be considered. First of all, the culture medium was only collected at day 3 of embryo culture. Whether collection of culture medium at another time point could also generate similar results may require further examination. Secondly, the number of clinical samples were limited, and in the future an increased number of clinical samples as determined by the power calculation should be considered to confirm the current findings. Thirdly, we did not examine whether the metabolic profiles related to the quality of embryos may be determined to further elucidate the predictive role of amino acids profiling in pregnancy outcome in IVF. In the study, we used both the peak height and surface area for the data analysis, while the peak height can be varied among different tests. In order to achieve more reliable prediction, we should optimize these parameters in more clinical samples.

## Conclusion

In conclusion, our results showed the significant differences in SER, ASP, HIS, and ALA concentrations in the spent culture media between embryos that resulted in pregnancy and embryos that failed to implant. Furthermore, the discrimination model which derived from the data applied for principal component analysis and discriminant analysis using amino acid data showed a good predictive accuracy for the pregnancy outcome. Although the current amino acid metabolism profile cannot be fully used as an independent reference, it can be used in conjunction with morphological assessment methods to increase accuracy in predicting embryo implantation potential.

## Data Availability Statement

All the data in the study is available upon request from the corresponding author.

## Ethics Statement

This study was approved by the Ethics Committee of Affiliated Hospital of Guilin Medical University. The patients/participants provided their written informed consent to participate in this study.

## Author Contributions

XL and SZ designed the whole study. PH and YZ performed all the experiments. JL, PH, YZ, and CL performed the data analysis. JY and LQ performed the statistical analysis. XL and SZ wrote the manuscript. All authors approved the manuscript for submission.

## Conflict of Interest

The authors declare that the research was conducted in the absence of any commercial or financial relationships that could be construed as a potential conflict of interest.

## References

[B2] Alpha Scientists in Reproductive Medicine and ESHRE Special Interest Group of Embryology (2011). Istanbul consensus workshop on embryo assessment: proceedings of an expert meeting. *Reprod. Biomed.* 22 632–646. 10.1016/j.rbmo.2011.02.001 21481639

[B3] American College of Obstetricians and Gynecologists Committee on Gynecologic Practice and Practice Committee (2014). Female age-related fertility decline. Committee Opinion No. 589. *Fertil. Steril.* 101 633–634. 10.1016/j.fertnstert.2013.12.032 24559617

[B4] Armstrong S. Bhide P. Jordan V. Pacey A. Farquhar C. (2018). Time-lapse systems for embryo incubation and assessment in assisted reproduction. *Cochrane Database Syst. Rev.* 5:Cd011320. 10.1002/14651858.CD011320.pub3 29800485 PMC6494546

[B5] Athayde Wirka K. Chen A. A. Conaghan J. Ivani K. Gvakharia M. Behr B. (2014). Atypical embryo phenotypes identified by time-lapse microscopy: high prevalence and association with embryo development. *Fertil. Steril.* 101 1637–1648.e1-e5. 10.1016/j.fertnstert.2014.02.050 24726214

[B6] Borowiecka M. Wojsiat J. Polac I. Radwan M. Radwan P. Zbikowska H. M. (2012). Oxidative stress markers in follicular fluid of women undergoing in vitro fertilization and embryo transfer. *Syst. Biol. Reprod. Med.* 58 301–305. 10.3109/19396368.2012.701367 22950633

[B7] Boyama B. A. Cepni I. Imamoglu M. Oncul M. Tuten A. Yuksel M. A. (2016). Homocysteine in embryo culture media as a predictor of pregnancy outcome in assisted reproductive technology. *Gynecol. Endocrinol.* 32 193–195. 10.3109/09513590.2015.1102877 26806445

[B8] Brison D. R. Houghton F. D. Falconer D. Roberts S. A. Hawkhead J. Humpherson P. G. (2004). Identification of viable embryos in IVF by non-invasive measurement of amino acid turnover. *Hum. Reprod.* 19 2319–2324. 10.1093/humrep/deh409 15298971

[B9] De Rose M. B. Piccolomini M. M. Soares Belo A. S. Borges E. Jr. Filho F. F. (2018). Proteomics in human reproduction. *Protein Pept. Lett.* 25 420–423. 10.2174/0929866525666180412164602 29651940

[B10] Egea R. R. Puchalt N. G. Escriva M. M. Varghese A. C. (2014). OMICS: current and future perspectives in reproductive medicine and technology. *J. Hum. Reprod. Sci.* 7 73–92. 10.4103/0974-1208.138857 25191020 PMC4150148

[B11] Fiehn O. (2002). Metabolomics–the link between genotypes and phenotypes. *Plant Mol. Biol.* 48 155–171. 10.1007/978-94-010-0448-0_11 11860207

[B12] Fragouli E. Alfarawati S. Spath K. Wells D. (2014). Morphological and cytogenetic assessment of cleavage and blastocyst stage embryos. *Mol. Hum. Reprod.* 20 117–126. 10.1093/molehr/gat073 24184690

[B13] Garlow S. J. (2002). And now, transcriptomics. *Neuron* 34 327–328. 10.1016/s0896-6273(02)00680-3 11988161

[B14] Gilchrist A. Au C. E. Hiding J. Bell A. W. Fernandez-Rodriguez J. Lesimple S. (2006). Quantitative proteomics analysis of the secretory pathway. *Cell* 127 1265–1281. 17174899 10.1016/j.cell.2006.10.036

[B15] Heijne W. H. Stierum R. H. Slijper M. Van Bladeren P. J. Van Ommen B. (2003). Toxicogenomics of bromobenzene hepatotoxicity: a combined transcriptomics and proteomics approach. *Biochem. Pharmacol.* 65 857–875. 10.1016/s0006-2952(02)01613-1 12628495

[B16] Kern A. Tilley E. Hunter I. S. Legisa M. Glieder A. (2007). Engineering primary metabolic pathways of industrial micro-organisms. *J. Biotechnol.* 129 6–29. 10.1016/j.jbiotec.2006.11.021 17196287

[B17] Li X. X. Cao P. H. Han W. X. Xu Y. K. Wu H. Yu X. L. (2018). Non-invasive metabolomic profiling of culture media of ICSI- and IVF-derived early developmental cattle embryos via Raman spectroscopy. *Anim. Reprod. Sci.* 196 99–110. 10.1016/j.anireprosci.2018.07.001 30001829

[B18] Liberman R. F. Getz K. D. Heinke D. Luke B. Stern J. E. Declercq E. R. (2017). Assisted reproductive technology and birth defects: effects of subfertility and multiple births. *Birth Defects Res.* 109 1144–1153. 10.1002/bdr2.1055 28635008 PMC5555800

[B19] Montsko G. Zrinyi Z. Janaky T. Szabo Z. Varnagy A. Kovacs G. L. (2015). Noninvasive embryo viability assessment by quantitation of human haptoglobin alpha-1 fragment in the in vitro fertilization culture medium: an additional tool to increase success rate. *Fertil. Steril.* 103 687–693. 10.1016/j.fertnstert.2014.11.031 25577461

[B20] Nadal-Desbarats L. Veau S. Blasco H. Emond P. Royere D. Andres C. R. (2013). Is NMR metabolic profiling of spent embryo culture media useful to assist in vitro human embryo selection? *Magma* 26 193–202. 10.1007/s10334-012-0331-x 22878530

[B21] Nel-Themaat L. Nagy Z. P. (2011). A review of the promises and pitfalls of oocyte and embryo metabolomics. *Placenta* 32 (Suppl. 3), S257–S263. 10.1016/j.placenta.2011.05.011 21703683

[B22] Rodgaard T. Heegaard P. M. Callesen H. (2015). Non-invasive assessment of in-vitro embryo quality to improve transfer success. *Reprod. Biomed.* 31 585–592. 10.1016/j.rbmo.2015.08.003 26380864

[B23] Scoccia B. (2015). What is new in assisted reproduction and multiple pregnancy reduction?: best articles from the past year. *Obstet. Gynecol.* 126 446–447. 10.1097/aog.0000000000000979 26241437

[B24] Scott L. A. Smith S. (1998). The successful use of pronuclear embryo transfers the day following oocyte retrieval. *Hum. Reprod.* 13 1003–1013. 10.1093/humrep/13.4.1003 9619562

[B25] Seli E. Botros L. Sakkas D. Burns D. H. (2008). Noninvasive metabolomic profiling of embryo culture media using proton nuclear magnetic resonance correlates with reproductive potential of embryos in women undergoing in vitro fertilization. *Fertil. Steril.* 90 2183–2189. 10.1016/j.fertnstert.2008.07.1739 18842260

[B26] Stokes P. J. Hawkhead J. A. Fawthrop R. K. Picton H. M. Sharma V. Leese H. J. (2007). Metabolism of human embryos following cryopreservation: implications for the safety and selection of embryos for transfer in clinical IVF. *Hum. Reprod.* 22 829–835. 10.1093/humrep/del447 17138583

[B27] Sturmey R. G. Brison D. R. Leese H. J. (2008). Symposium: innovative techniques in human embryo viability assessment. Assessing embryo viability by measurement of amino acid turnover. *Reprod. Biomed.* 17 486–496. 10.1016/s1472-6483(10)60234-9 18854101

[B28] Wu G. (2010). Functional amino acids in growth, reproduction, and health. *Adv. Nutr.* 1 31–37. 10.3945/an.110.1008 22043449 PMC3042786

[B29] Zhou Z. Zheng D. Wu H. Li R. Xu S. Kang Y. (2018). Epidemiology of infertility in China: a population-based study. *BJOG* 125 432–441. 10.1111/1471-0528.14966 29030908

